# Therapeutic potential of Janus kinase inhibitors for the management of fibrosis in inflammatory bowel disease

**DOI:** 10.1093/ecco-jcc/jjaf087

**Published:** 2025-05-24

**Authors:** Jie Su, Dalia A Lartey, Gaia Zanella, Lukas J A C Hawinkels, Gianluca Matteoli, Mark Löwenberg, Marieke C Barnhoorn

**Affiliations:** Department of Gastroenterology and Hepatology, Leiden University Medical Center, Leiden, The Netherlands; Department of Gastroenterology and Hepatology, Amsterdam UMC, Amsterdam, The Netherlands; Translational Research Center for Gastrointestinal Disorders (TARGID), KU Leuven, Leuven, Belgium; Department of Gastroenterology and Hepatology, Leiden University Medical Center, Leiden, The Netherlands; Translational Research Center for Gastrointestinal Disorders (TARGID), KU Leuven, Leuven, Belgium; Department of Gastroenterology and Hepatology, Amsterdam UMC, Amsterdam, The Netherlands; Department of Gastroenterology and Hepatology, Leiden University Medical Center, Leiden, The Netherlands

**Keywords:** Janus kinase, JAK, JAK-STAT pathway, JAK inhibitors, fibrosis, inflammatary bowel disease, IBD, Crohn’s disease, stenosis

## Abstract

Intestinal fibrosis in inflammatory bowel disease (IBD) is caused by uncontrolled accumulation of extracellular matrix deposited by fibroblasts. This may result in stricture formation, especially in Crohn’s disease. Since there are no anti-fibrotic drugs available, endoscopic or surgical interventions are the only options to treat intestinal strictures. The Janus kinase-signal transducer and activator of transcription (JAK-STAT) pathway plays a crucial role in intestinal homeostasis and inflammation. JAK inhibition represents a relatively novel therapeutic strategy in IBD by simultaneously blocking multiple cytokines across various inflammatory pathways. Interestingly, JAK inhibitors extend their benefits beyond anti-inflammatory effects, as they have been shown to interfere with fibrotic processes in various diseases, including IBD. We here summarize the current understanding of the role of the JAK-STAT pathway in the pathogenesis of intestinal fibrosis and the application of JAK inhibitors for IBD. In addition, we discuss the use of JAK inhibitors in other fibrotic-related diseases to postulate how these agents might be applied for future treatment of intestinal fibrosis.

## 1. Introduction

Intestinal fibrosis is a common complication of inflammatory bowel disease (IBD), affecting approximately 50% of patients with Crohn’s disease (CD) and around 5% of those with ulcerative colitis (UC).^1^ Fibrosis formation, marked by excessive accumulation of extracellular matrix (ECM) components, is a normal and crucial phase of tissue repair in all organs. However, in diseases like IBD, this process becomes excessive and uncontrolled in a substantial proportion of patients, leading to increased tissue stiffness, stenosis formation, and malfunction.^2^ In CD, fibrosis can affect all layers of the bowel wall, while in UC, it impacts the mucosa and submucosa.^3^ In CD patients, fibrosis can become symptomatic by the development of fibrostenotic strictures, leading to narrowing of the bowel lumen. Patients with symptomatic bowel strictures can be treated by endoscopic balloon dilatation, which is effective in up to 40% of patients, and often has to be repeated. Eventually 75% of such patients will need surgery.^4^ Currently, no anti-fibrotic pharmacological therapies are available for IBD. In order to develop anti-fibrotic agents, a deeper understanding of the mechanisms underlying fibrosis formation and progression in IBD is essential. The lack of standardized methodology to score intestinal fibrosis is hampering the clinical development of potential anti-fibrotic agents. Recently, researchers from the STAR (stenosis therapy and anti-fibrotic research) consortium presented a consensus statement where they agreed that cross-sectional imaging, using magnetic resonance imaging, computed tomography (CT), or intestinal ultrasound, in addition to inability to pass the affected area with a colonoscope, is appropriate to diagnose fibrostenotic CD, but not to determine the degree of fibrosis.^5^ Currently, novel molecular imaging techniques, like the fibroblast activation protein inhibitor **Positron Emission Tomography (PET)**/CT scan, are being evaluated for their capability to score the degree of fibrosis and to differentiate this from active inflammation in IBD patients.^6^

Inflammation has always been regarded as one of the main drivers for fibrogenesis. During intestinal inflammation, fibroblasts play a critical role by responding to cytokines and growth factors released by both immune and non-immune cells. This leads to ECM production and tissue remodeling, which is crucial for the initiation and progression of intestinal fibrosis formation.^7^ The Janus kinase-signal transducer and activator of transcription (JAK-STAT) pathway is a cellular signaling pathway activated in response to inflammatory cytokines, such as interleukins (IL-6, IL-2, IL-12, IL-22, IL-23, IL-10, and interferons [IFNs]), but also erythropoietin, thyroperoxidase, granulocyte colony-stimulating factor, growth hormone, and leptin. This pathway regulates a wide range of biological processes such as proliferation, differentiation, migration, apoptosis, and cell survival of various cell types, influencing the process of inflammation and fibrosis by causing changes in DNA transcription.^8,9^ Upon binding of a ligand, the intracellular JAK proteins (JAK1, JAK2, JAK3, and tyrosine kinase 2 [TYK2]) dimerize, leading to phosphorylation of JAKs and subsequent activation of STATs.^10,11^ Phosphorylated STATs subsequently dimerize and translocate into the nucleus, thereby regulating the transcription of associated genes.^12^ Initially, activated JAK-STAT pathways were primarily found in immune cells, but later also described in other cell types like fibroblasts, endothelial cells, and epithelial cells.^13–15^

Janus kinase inhibitors are a relatively novel class of drugs approved for IBD with the advantages of oral administration, rapid onset of action, quick clearance, and lack of immunogenicity.^16^ Furthermore, compared to biologics such as tumor necrosis factor (TNF) inhibitors or anti-α4β7 integrin inhibitors, which target a single molecule, JAK inhibitors exhibit a robust anti-inflammatory capability by simultaneously blocking multiple cytokines across various inflammatory pathways. In Europe, filgotinib, upadacitinib (both preferential JAK1 inhibitors), and tofacitinib (pan-JAK inhibitor mainly targeting JAK1 and JAK3) have been approved for UC, and upadacitinib is available for CD.^17,18^ However, JAK inhibitors extend their benefits beyond anti-inflammatory effects, as they have also been shown to interfere with fibrotic processes in various diseases.^19^ Ruxolitinib, a selective inhibitor of JAK1/2, was the first Food and Drug Administration (FDA) and European Medicines Agency (EMA) approved therapy for myelofibrosis (MF) and has revolutionized the management of this disease. Furthermore, ruxolitinib treatment also impaired proliferation, migration, and activation of hepatic stellate cells (HSCs), a key player in driving liver fibrosis.^20^ Given these findings, we speculate that JAK inhibitors might not only play a role in IBD by reducing inflammation, but might also offer therapeutic potential to prevent or treat intestinal fibrosis.

In this review, we will summarize the current knowledge on the JAK-STAT pathway in the intestine and in fibrotic-related diseases beyond the gastrointestinal tract. Finally, we will provide an overview of available preclinical and clinical data of JAK-STAT pathway involvement in intestinal fibrosis in IBD.

## 2. JAK-STAT pathway in intestinal fibrosis

### 2.1. Key players in intestinal fibrosis

**Figure 1.**
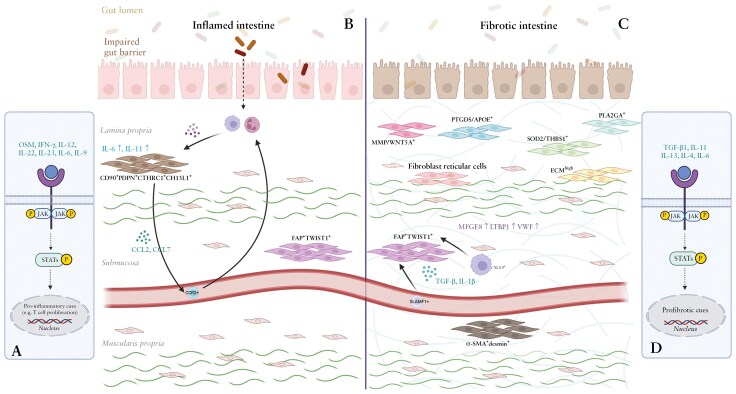
**Fibroblasts subsets and the JAK-STAT pathway in the pathogenesis of fibrostenotic Crohn’s disease**. (A) Cytokines that signal through the JAK-STAT pathway in inflamed CD. (B) Intestinal fibroblasts subsets that are identified in inflamed CD and play a potential role in fibrosis initiation are depicted. The CD90 ^+^ PDPN ^+^ CTHRC1 ^+^ CHI3L1 ^+^ fibroblast population was found to be associated with anti-TNF resistance in the inflamed intestine of CD patients.^22^ These subsets express profibrotic IL-11 and IL-6, next to monocyte-recruiting CCL2 and CCL7. Also, high levels of FAP ^+^ TWIST1 ^+^ fibroblasts were found in inflamed CD intestines.^23^ (C) High levels of ECM components VWF, MFGE8, and LTBP1 are found in fibrostentic CD.^21^ Different fibroblast subsets have been identified to play a role in fibrostenotic CD, including FAP ^+^ TWIST1^+^, α-SMA ^+^ desmin^+^, SOD2/THBS1^+^, PLA2GA^+^, PTGDS/APOE^+^, MMP/WNT5A^+^, fibroblast reticular cells, and ECM ^high^.^23-25^ (D) Cytokines that signal through the JAK-STAT pathway in stenotic CD. TWIST1, Twist-related protein 1; α-SMA, α-smooth muscle actin; CCR2, C-C chemokine receptor type 2; CHI3L1, chitinase-3-like protein 1 fibroblasts; CTHRC1, collagen triple helix repeat containing 1; CXCL, chemokine (C-X-C motif) ligand; ECM, extracellular matrix; FAP, fibroblast activation protein α; IFN-γ, interferon γ; IL, interleukin; LTBP1, latent transforming growth factor-β binding protein 1; MFGE8, milk fat globule-epidermal growth factor 8; MMP, matrix metalloproteinases; OSM, oncostatin-M; PDPN, podoplanin; SLAMF1, Signaling lymphocytic activation molecule 1; TGF-β, transforming growth factor beta; VWF, von Willebrand Factor.. Created in BioRender. Barnhoorn, M. (2025) https://BioRender.com/eq0lcb1

Before discussing the potential of JAK inhibitors to treat intestinal fibrosis, it is crucial to understand the underlying pathogenesis. Intestinal fibrosis is characterized by excessive accumulation of ECM together with increased smooth muscle layer thickness. In healthy conditions, the ECM is remodeled by matrix metalloproteinases (MMPs), capable of degrading ECM components and tissue inhibitors of MMPs (TIMPs) that inhibit MMP activity. Evaluation of the accumulated ECM in fibrostenotic ileal CD shows higher relative abundance of latent transforming growth factor (TGF)-β binding protein 1, milk fat globule-epidermal growth factor 8, and von Willebrand factor in comparison to inflamed tissue.^21^ Key cell types in the pathogenesis of intestinal fibrosis are ECM-producing stromal cells and more specifically fibroblasts and smooth muscle cells (summarized in Figure 1 and definitions described in Box 1). Recent advancements in single-cell omics revealed the substantial heterogeneity of these cells. In CD, the importance of stromal cells was elucidated with the recognition of a specific subset of fibroblasts in inflamed tissue, defined as CD90^+^podoplanin(PDPN)^+^collagen triple helix repeat containing 1 (CTHRC1)^+^chitinase-3-like protein 1 (CHI3L1)^+^ fibroblasts. These fibroblasts produce, among others, monocyte attraction molecules like C-C motif chemokine ligand 2 and CCL7, and are part of a cellular profile associated with anti-TNF resistance in ileal CD.^22^ Interestingly, these cells also showed increased expression of IL-6 and IL-11, both ILs associated with fibrosis. Furthermore, in inflamed CD ileum gremlin (GREM)^+^CD34^+^ fibroblasts, FAP^+^ fibroblasts, contractile pericytes, and myofibroblasts are overrepresented compared to uninflamed ileum.^23^ In fibrostenotic CD, a specific fibroblast population defined as FAP^+^Twist-related protein 1 (TWIST1)^+^ was recently discovered to be predominantly responsible for extensive ECM production.^21,23^ Specific inflammatory SLAMF1^+^ (Signaling lymphocytic activation molecule 1) monocytes and C-X-C motif ligand (CXCL)9^+^macrophages were found to be especially important in the activation of these fibroblasts via IL-1β and TGF-β signaling. Another single-cell (sc)RNA sequencing dataset using ileal samples from CD patients suffering from stenosis showed overexpression of 6 different fibroblast populations, including MMP/WNT5A^+^ fibroblasts, compared to inflamed and uninflamed tissue. All of these fibroblast populations showed expression of markers involved in TGF-β regulation and ECM production, thereby indicating their importance for fibrosis development.^24^

Traditionally, myofibroblasts, defined as α-smooth muscle actin (α-SMA)^+^ fibroblasts, have been regarded as main players in fibrosis, since they secrete extensive ECM proteins in vitro. Interestingly, current scRNA sequencing data do not fully support this theory since other fibroblast subsets like the FAP-expressing fibroblasts seem to be a more abundant source of ECM production. Interestingly, in most in vitro experiments, α-SMA expression is still regarded as a hallmark of fibroblast activation. Previously, fibroblast-to-myofibroblast transition and subsequent upregulation of α-SMA (*ACTA2*) together with increased proliferation and ECM production were defined as fibroblast activation. In the current review, we define activated fibroblasts as fibroblasts that are proliferating and producing high levels of ECM molecules. Of note, smooth muscle cells, as another stromal cell subtype, defined as α-SMA^+^ and desmin^+^ cells, are also important players in fibrostenosis since their hypertrophy and hyperplasia lead to thickening of the muscularis propria and they also contribute to ECM production. Interestingly, current research on fibrostenosis is mainly focused on ECM production and less on smooth muscle cell hyperplasia and hypertrophy.^25^ Although stromal cells are the main producers of ECM, the interplay between immune cells and stromal cells is crucial for the process of fibrostenosis. A more comprehensive list of profibrogenic mediators in gut fibrosis was recently published,^26^ including IL-1, IL-4, IL-13, IL-17, TNF-like ligand 1A, IL-11, IL-33, and many more. For this review, it is important to mention that part of these cytokines signal through the JAK-STAT pathway, which we will discuss in paragraph 2.2.

Box 1: **Definitions of stromal cell subsets in intestinal fibrosis**

**Definitions**

*Fibroblast*: subset of stromal cells defined by a combination of their morphology, tissue position, and lack of lineage markers for epithelial cells, endothelial cells, and leukocytes.^27^ Commonly used markers are platelet-derived growth factor receptor (PDGFR)-α and vimentin, but typically alongside other criteria since there is not one exclusive fibroblast marker available yet.

*Myofibroblast*: α-SMA expressing fibroblast with contractile features.

*Activated fibroblast*: in the current review, we define activated fibroblasts as fibroblasts that are proliferating and producing high levels of ECM molecules.

### 2.2. JAK-STAT pathway and intestinal fibrosis

The JAK-STAT pathway is an essential pathway for gut homeostasis and has been shown to coordinate epithelial regeneration and homeostatic immune responses.^28^ For example, the importance of JAK3 in the regulation of intestinal homeostasis was shown by the development of spontaneous colitis in JAK3 gene-disrupted mice.^29^ Already in homeostatic conditions, low-level expression of STAT1 and STAT2 target genes was found in resting (immune) cells in several studies.^30,31^ In contrast to the subtle role of the JAK-STAT pathway in homeostatic conditions, its role is more clearly present in acute and chronic inflammation of the intestine. By regulating both innate and adaptive mucosal immunity, the JAK-STAT pathway is directly involved in the inflammatory process and clearance of pathogens, summarized in several excellent reviews.^32,33^ In the context of IBD, important proinflammatory cytokines like oncostatin-M, IFN-γ, IL-12, IL-22, IL-23, IL-6, IL-9 signal via the JAK-STAT signaling pathway. Genome-wide association studies indicated that genetic risk loci in the JAK-STAT pathway are associated with IBD.^34^ Especially *JAK2* “gain of function” variants have been linked to both UC and CD. Furthermore, associations between IBD and *TYK2/STAT3/4/6* gene variants were described. In both human intestinal samples from IBD patients and different in vivo murine IBD models, activation of the different STATs has been confirmed especially in T cells and macrophages.^35^ Interestingly, in IBD, JAK-STAT activation can play dual roles depending on the cell type in which it is activated. In general, activation of the JAK-STAT pathway in immune cells leads to inflammatory responses, but activation of the pathway by IL-22 in human colonic organoids increased epithelial regeneration.^14^ In line with these results, the JAK inhibitor tofacitinib impaired DSS-induced colitis epithelial cell recovery showing the importance of JAK signaling for initiating epithelial regeneration in vivo.^14^ In accordance with these results, it was shown that enterocyte-specific *STAT5* or *STAT3* deletion increases the severity of DSS-induced colitis.^36,37^ Further data on cell-specific effects of JAK inhibitors are described in this recent review.^38^

The suggestion that the JAK-STAT pathway could be directly involved in fibrogenesis was raised by the association of specific JAK2 SNPs in IBD patients, with ileocolonic disease and increased risk of stricturing complications.^39–41^ Fibroblasts, and especially activated fibroblast, are the main cells responsible for excessive ECM deposition and therefore inducing fibrosis. Many of the cytokines that are profibrogenic signal through the JAK-STAT pathway, such as cytokines from the IL-6 family including IL-11 (JAK1/2, TYK2/STAT3), IL-13, and IL-4 (JAK1/3/STAT5).^26,42^ Transforming growth factor-β1 is regarded as one of the most potent profibrotic cytokines and although it primarily signals through the SMAD2/3 signaling complex, there is crosstalk with the JAK-STAT pathway. For example during hepatic fibrosis, it has been shown that TGF-β can directly activate the JAK1-STAT3 pathway.^43^ Furthermore, activation of the JAK-STAT pathway can be induced by biophysical forces that are often present in fibrostenosis, through cell-matrix interactions and ECM stiffness.^44^

In patients with fibrostenotic CD, STAT3 activation has been shown and seems to be associated with TGF-β1 upregulation in these patients. STAT3(Y705) and STAT3(S727) have been found to be the most functionally relevant phosphorylation sites (p) of STAT3. In smooth muscle cells isolated from CD-related intestinal strictures, pSTAT3(Y705) was lower, while pSTAT3(S727) was higher compared to unaffected parts of the intestine. Interestingly, it was shown that pSTAT3(S727) and not pSTAT3(Y705) regulated TGF-β1 DNA-binding activity, resulting in increased production of TGF-β1 and COL1.^45^ Inhibition of STAT3 phosphorylation in cultured dermal fibroblasts showed prevention of differentiation of fibroblasts into myofibroblasts, inhibition of α-SMA upregulation, formation of stress fibers, and lower expression of COL1.^46,47^ Recently, TWIST1 has been described by our and other research groups as key regulator of the activation process of FAP^+^ fibroblasts in stenotic CD patients.^23,48^ Interestingly, in other diseases, TGF-β1 has been described to directly regulate the expression of TWIST1 via pSTAT3.^49,50^ Furthermore, upregulation of IL-11, member of the JAK-STAT signaling IL-6 family, together with activation of STAT3 was observed in these FAP^+^ fibroblasts that were isolated from stenotic ileum segments of CD patients,^23,51^ showing once again the importance of JAK-STAT signaling in fibrotic CD. A very recent article using longitudinal scRNA sequencing of both immune and non-immune cell populations from colonic biopsies of UC patients treated with the JAK inhibitor tofacitinib further confirms the involvement of these pathways in driving the crosstalk between myeloid and stromal cells.^52^ Limited preclinical studies have been performed with JAK-STAT inhibition to prevent intestinal fibrosis. pSTAT3 inhibition in mice with 2,4,6-trinitrobenzenesulfonic acid-induced colitis showed no statistically significant decrease in muscularis propria thickness and collagen deposition.^45^ However, mRNA expression of *TGF-β1* and *COL1A1* was significantly reduced in the colon of mice treated with the pSTAT3 inhibitor. In a Th17 cell transfer model for IBD, treatment of mice with pSTAT3 inhibitors reduced the production of collagen proteins, such as COL1A1, COL6A1, and αSMA, resulting in decreased fibrosis.^53^

Hence, there is accumulating evidence for an important role of especially JAK2-STAT3 in the pathogenesis of intestinal fibrosis, and preliminary results show that JAK inhibitors could directly affect fibroblasts, which are key cells in driving fibrosis (Figure 1).

## 3. JAK-STAT pathway in fibrotic diseases

### 3.1. Myelofibrosis

**Figure 2.**
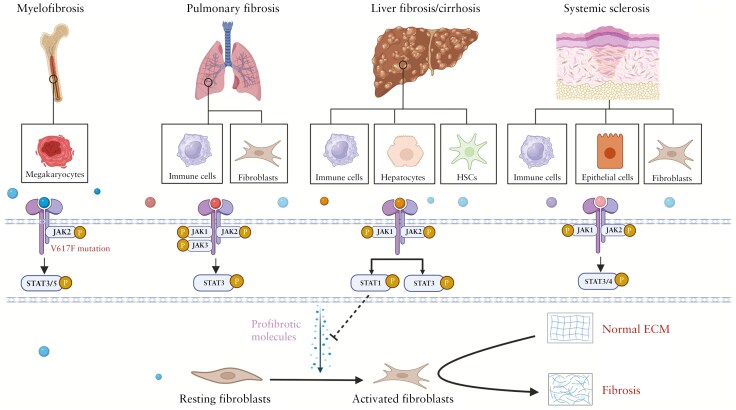
JAK-STAT pathways are involved in different cell types in several fibrotic diseases. Top: the different cell types that signal through JAK-STAT are involved in myelofibrosis, pulmonary fibrosis, liver fibrosis/cirrhosis, and systemic sclerosis. Middle: main JAKs and STATs involved in the different fibrotic diseases. Bottom: the general process of fibrosis in which activated fibroblasts lead to excessive ECM deposition and dysregulated ECM remodeling. ECM, extracellular matrix; HSC, hepatic stellate cells; JAK, Janus kinase; STAT, signal transducer and activator of transcription proteins. Created in BioRender. Barnhoorn, M. (2025) https://BioRender.com/sxtkq4w

Myelofibrosis is a progressive myeloid disorder characterized by the deposition of reticulin, causing fibrosis and an increased risk of leukemic transformation.^54^ The pivotal role of the JAK-STAT signaling pathway in MF pathogenesis was elucidated through the identification of the *JAK2-V617F* mutation in 50%-60% of MF patients.^55^ This mutation disrupts the autoinhibitory SH2 pseudokinase domain of JAK2, leading to constitutive activation and subsequent STAT-mediated transcriptional upregulation. Patients harboring the JAK2-V617F mutation typically exhibit a worse prognosis compared to those with wild-type *JAK2*.^55,56^ Recent in vivo studies in megakaryocytes reveal that hyperactive JAK-STAT signaling is a driver of sustained myeloproliferation via cytokine release and thereby impacts non-clonal hematopoietic components within the bone marrow.^57^ JAK-STAT activation in both malignant and nonmalignant hematopoietic cells has been linked to abnormal proinflammatory cytokine production.^58^ Additionally, non-hematopoietic cell populations in the bone marrow, such as nestin^+^ stromal cells, contribute to disease progression.^59^ STAT3, alongside JAK2, plays a pivotal role in cytokine production in MF (Figure 2); its deletion across all hematopoietic elements mitigates cytokine overproduction, reduces disease severity, and improves survival.^58^ These discoveries have paved the way for the development of JAK-STAT pathway targeted therapies, with 2 such strategies already approved for MF treatment. Ruxolitinib, a selective inhibitor of JAK1 and -2, revolutionized the landscape of MF treatment by being the first FDA-approved targeted therapy for this condition and the first JAK inhibitor on the market.^60^ Nevertheless, many patients discontinue the drug within a few years mainly due to the development of cytopenia. The prognosis of patients with MF who cease ruxolitinib is poor, with median survival ranging from 6 to 14 months.^61–63^ Fedratinib, another JAK2 inhibitor may fulfill an important unmet need for MF patients with resistance or intolerance to ruxolitinib, because ruxolitinib-resistant JAK2 variants show little or no resistance to fedratinib.^64^ Despite clinical improvement by JAK inhibitors, the first studies did not reveal any substantial treatment impact on bone marrow histomorphology, like fibrosis. However, long-term ruxolitinib therapy showed delayed progression of bone marrow fibrosis.^65^ Based on these observations, the introduction of JAK inhibitors represents a significant advancement in the management of MF and shows initial signs for inhibition of fibrotic processes.

### 3.2. Pulmonary fibrosis

Interstitial lung diseases (ILDs) encompass a group of disorders characterized by lung injury as a result of fibroblast proliferation, interstitial inflammation, and fibrosis.^66^ Interstitial lung diseases can originate from many etiologies including medication, infections, radiation-induced lung diseases, and autoimmune diseases such as systemic sclerosis (SSc) and rheumatoid arthritis (RA).^67^ Idiopathic pulmonary fibrosis is a specific subtype of ILD characterized by progressive scarring (fibrosis) of the lung tissue without a known cause.^66,68^ The JAK-STAT signaling pathway plays a significant role in the pathogenesis and progression of pulmonary fibrosis by promoting inflammation and fibrotic signaling. This fibrotic signaling via ILs but also TGF-β causes fibroblast activation, but also epithelial-to-mesenchymal transition and induction of cellular senescence via the JAK-STAT pathway in ILD. Overexpression of cytokines such as IL-4, IL-6, IL-13, and TGF-β1 in ILD activates the JAK-STAT pathway, leading to macrophage activation with subsequent increased release of proinflammatory and profibrotic factors, and the differentiation of fibroblasts into myofibroblasts (Figure 2).^69^ Janus kinase-signal transducer and activator of transcriptions have indeed been found to be overexpressed in lung tissues of patients with ILD. More specifically, JAK2-STAT3 is considered to play a key role in ILD development.^70^ Systemic sclerosis and ILD patients showed higher phosphorylation of JAK1/2/3 and STAT3 compared to healthy controls both in skin and lung biopsy.^71^ In animal models, STAT3 has been demonstrated to regulate the differentiation of lung fibroblasts into myofibroblasts by IL-6 and TGF-β1.^72^ In a mouse model for autoimmune pulmonary fibrosis, treatment with the JAK1 inhibitor ruxolitinib prevented the upregulation of proinflammatory markers in M1 macrophages and profibrotic markers in M2 macrophages, leading to improvement in lung inflammation and fibrotic lesions.^73^ Moreover, another study reported that intraperitoneal injection of tofacitinib inhibited the progression of ILD in a monogenic model of autoimmune arthritis due to altered signal transduction in T cells by facilitating the expansion of myeloid suppressor cells in the lungs.^74^ Short-term tofacitinib effectively blocked IL-6-induced fibroblast activation and fibrosis in bleomycin-induced pulmonary fibrosis model. Interestingly, the anti-fibrotic effects of selective JAK2 inhibitors on fibroblasts and in models for pulmonary fibrosis decreased over time because of reactivation of JAK2 by induction of JAK1.^75^ In human studies, a single-arm open-label trial of 18 patients revealed that tofacitinib demonstrates greater survival in patients with amyopathic dermatomyositis-ILD compared to conventional treatment.^76^ A retrospective study evaluated the efficacy of tofacitinib in patients with dermatomyositis-ILD resistant to triple therapy (high-dose glucocorticoid, cyclosporine A, and cyclophosphamide). The results of this study showed that the patients treated with tofacitinib had a higher survival rate than patients in the pre-JAK inhibition era.^77^ Both studies suggested that tofacitinib is a potential new treatment for dermatomyositis-ILD patients. Furthermore, a few case reports described the use of JAK inhibitors in ILDs related to other autoimmune diseases (like STING-associated vasculopathy and systemic idiopathic juvenile arthritis).^78,79^ However, most human clinical data on JAK inhibition in ILD come from RA patients with ILD, since JAK inhibition was approved for RA first. The Janus kinase-signal transducer and activator of transcription pathway inhibition in RA-ILD patients resulted in improved pulmonary functions and reduced proinflammatory cytokines like IL-6.^80^ Moreover, combination therapies of JAK inhibition and T cell inhibition using abatacept, a drug inhibiting CD80/86 and thereby T cell activation, showed slower ILD progression in patients with RA-ILD.^81^ Although these findings suggest that JAK inhibition might be efficacious to treat fibrosis-related ILD, large-scale randomized controlled trials are needed to confirm this.

### 3.3. Liver cirrhosis

Liver fibrosis is triggered by various liver diseases such as hepatitis B virus, metabolic dysfunction-associated steatohepatitis, and alcoholic liver disease. Prolonged fibrosis can eventually lead to irreversible liver cirrhosis. During the progression of fibrosis, inflammation and liver injury initiate complex cellular processes that lead to collagen deposition and the disruption of normal liver architecture.^82^ Activation and differentiation of HSCs into activated fibroblast are crucial events in liver fibrogenesis.^83^ Additionally, immune cells may regulate fibrogenesis via the secretion of a wide variety of growth factors and cytokines.^84^ Many of these cytokines, such as IL-6, IFN-γ, IFN-α/β, and IL-22, can activate the JAK-STAT signaling pathway in the liver (Figure 2).^85^ Numerous studies have investigated the role of JAK-STAT in hepatic fibrosis, but the findings are not consistent. In the majority of studies, activation of STAT1 is considered beneficial to prevent fibrosis. Proteomic analysis of activated HSCs identified STAT1 as a crucial regulator in the transition between liver fibrosis and recovery.^86^ STAT1 attenuated liver fibrosis by inhibiting HSCs proliferation, reducing TGF-β signaling, and enhancing natural killer cell-mediated killing of activated HSCs.^87^ In the carbon tetrachloride (CCL4)-induced hepatic fibrosis mouse model, STAT1 knockout mice exhibited significantly faster disease progression compared to the control group.^87^ Next to STAT1, also STAT3 is playing a crucial role in liver fibrosis. However, conditional inactivation of STAT3 in hepatocytes and cholangiocytes in a murine model for sclerosing cholangitis significantly worsened bile acid-induced liver injury and fibrosis.^88^ Furthermore, STAT3-dependent signaling pathways in hepatocytes protected against apoptosis and tissue damage, which in turn helped to prevent the progression of fibrosis.^89^ In contrast, high STAT3 phosphorylation was shown in liver cirrhosis samples, and in vitro experiments showed that STAT3-mediated signaling upregulated TGF-β1 expression in hepatocytes.^90^ Despite these conflicting findings, the STAT3 inhibitor S3I-201 showed great potential in the CCL4-induced hepatic fibrosis mouse model.^91^ Furthermore, S3I-201 was capable of inhibiting the proliferation, migration, as well as the expression of α-SMA, COL1, and TIMP1 in primary HSCs.^91^ Along the same lines, another study showed that activation of the JAK1-STAT3 signaling axis promoted hepatic fibrosis in conjunction with the TGF-β/SMAD pathway.^43^ Currently, JAK inhibitors are not approved for clinical use to treat liver fibrosis or cirrhosis. However,  they are presently being evaluated in both preclinical and clinical research settings. The JAK2 inhibitor pacritinib was found to decrease collagen deposition in CCL4/ethanol‐induced mouse model for liver fibrosis and suppresses proliferation, contraction, and migration of HSCs in vitro.^92^ Moreover, the JAK1/2 inhibitor ruxolitinib reduces the proliferation, migration, and activation of HSCs in vitro and accelerates the reversal of liver fibrosis in different murine models.^20^ A study using an autoimmune hepatitis mouse model demonstrated attenuation of liver fibrosis after treatment with tofacitinib by altering the disbalance between Tregs and Th17 cells.^93^ The only clinical data available are from a study in which patients with both IBD and primary sclerosing cholangitis (PSC) were treated with tofacitinib. This study showed a significant decline of alkaline phosphatase serum levels, a marker for the activity and prognosis of PSC in treated patients.^94^ In conclusion, although the definite role of the JAK-STAT pathway in liver fibrosis is not established, and probably depends on disease state and cell type, most in vivo results on JAK inhibition are promising. Therefore, it seems the right time to start a clinical trial using JAK inhibitors in patients with liver fibrosis. Furthermore, it would be especially valuable to study IBD patients with concomitant PSC with signs of hepatic fibrosis receiving treatment with JAK inhibitors with sufficient follow-up.

### 3.4. Systemic sclerosis

Systemic sclerosis , also known as scleroderma, is a fibrotic autoimmune disorder with a high morbidity and mortality and notably characterized by fibrosis of the skin and internal organs as well as vasculopathy.^95^ Key pathological processes include endothelial damage, fibroblast activation, and excessive deposition of ECM, all resulting in uncontrolled tissue fibrosis. While significant advances have been made in the treatment of SSc in recent years, effective disease-modifying therapies are still lacking. The JAK-STAT signaling pathway was reported to play a crucial role in proinflammatory and profibrotic signaling in inflammatory cells, epithelial cells, and fibroblasts in SSc.^71^ Increased activation of JAK2 was detected in the skin of patients with SSc, particularly in fibroblasts (Figure 2).^96^ Inactivation of the transcription factor STAT4 prevented inflammation-driven fibrosis in animal models of SSc.^97^ Furthermore, increased levels of activated STAT3 were observed in skin biopsies from a murine SSc model.^98^ These data imply that the JAK-STAT pathway could be a target for the treatment of SSc. Several inhibitors of the JAK-STAT pathway have been tested in preclinical and clinical studies. The JAK2 inhibitor TG101209 was reported to not only prevent bleomycin-induced fibrosis, but also significantly reduce skin fibrosis in mice in a TGF-β-dependent manner.^96^ Inhibition of STAT3 with C188-9 reduced myofibroblast accumulation, profibrotic gene expression, collagen, and skin fibrosis in vivo. C188-9 also suppressed the production of fibrotic genes by dermal fibroblasts in vitro, which were induced by IL-6 and TGF-β.^98^ In the bleomycin-induced fibrosis model, ruxolitinib, tofacitinib, and baricitinib showed the capability to effectively reduce skin and/or lung fibrosis.^99–101^ Baricitinib also showed properties in 10 SSc patients to relieve skin fibrosis and finger/toe ulcers.^102^ Next to this report, a few other studies have evaluated the effects of JAK inhibitors in SSc patients.^103–105^ Retrospective studies on the effect of tofacitinib in SSc-ILD patients demonstrated significant improvements in serum inflammation markers, skin sclerosis, and the progression of pulmonary fibrosis.^106^ In the TOFA-SSc trial, a phase I/II double-blind, placebo-controlled trial, the safety and tolerability of tofacitinib were evaluated in SSc patients at week 24 and showed that tofacitinib was well tolerated. Additionally, tofacitinib inhibited the expression of IFN-regulated biomarker genes in skin fibroblasts and keratinocytes.^107^ Although the results from the preclinical and clinical studies are promising, phase III randomized controlled trial studies are required to assess the efficacy and toxicity of selective JAK inhibition in SSc patients. Since a considerable proportion of SSc patients also have gastrointestinal involvement, evaluation of gastrointestinal symptoms and intestinal samples before and after treatment with JAK inhibitors could be relevant for the treatment of intestinal fibrosis.

## 4. JAK inhibitors in IBD and potential anti-fibrotic effects

### 4.1. JAK inhibitors in IBD

**Figure 3.**
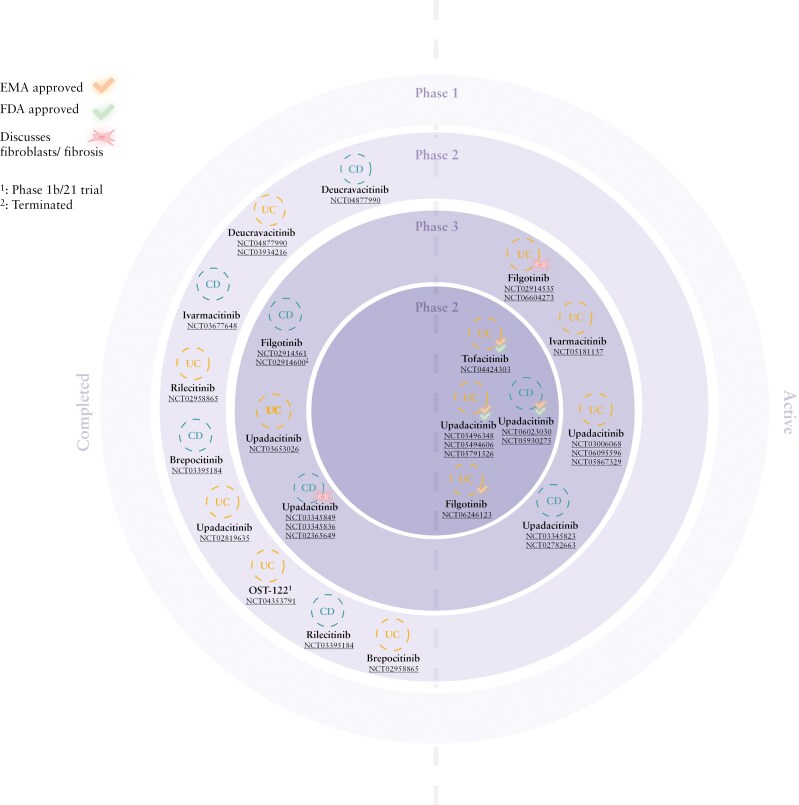
**Overview of active and completed clinical trials investigating JAK inhibitors in CD and UC.** It is indicated if a drug is EMA/FDA-approved and if fibroblasts are discussed in the paper. CD, Crohn’s disease; EMA, European Medicines Agency; FDA, Food and Drug Administration; JAK, Janus kinase; UC, ulcerative colitis. Created in BioRender. Barnhoorn, M. (2025) https://BioRender.com/5yvqr6x

Despite the different cell-specific roles of the JAK-STAT pathway in IBD, JAK inhibitors were successfully introduced for the treatment of IBD patients (Figure 3).^17,18^ Currently, tofacitinib, filgotinib, and upadacitinib are approved for the treatment of UC and upadacitinib for CD in Europe. Unfortunately, all these clinical trials excluded patients with stricturing CD, representing essentially patients with clinically relevant fibrosis. The study design of clinical trials investigating potential anti-fibrotic effects of JAK inhibitors is challenging mainly due to the lack of a standardized methodology to score the degree of intestinal fibrosis. For now, no cohort studies are available that looked into the ability of JAK inhibitors to prevent the development of intestinal fibrosis as this requires long-term follow-up studies (>5 years). Some insights into potential anti-fibrotic effects of JAK inhibitors for treating intestinal fibrosis can be extracted from the RNA sequencing data obtained from the CELEST trial, and from scRNA sequencing data of biopsies from patients treated with tofacitinib. The CELEST trial was a phase 2 induction and maintenance study which assessed the safety and efficacy of upadacitinib in CD patients. Endoscopic remission was highest in the high-dose group (22%) compared to placebo (0%, *P* < .01).^108^ A sub-study of CELEST assessed the underlying mechanisms of upadacitinib-dependent JAK1 inhibition in CD. To this end, ileal and colonic biopsies (at week 0 and weeks 12-16) were analyzed for gene expression using bulk RNA sequencing and aligned with publicly available single-cell data in order to assign transcriptional changes to a specific subset.^109^ In the colon and ileal mucosa, the most significantly regulated genes were expressed by enterocytes and myeloid/stromal cells, respectively. Overexpression of fibroblast-expressed genes in the colon, including *CHI3L1*, *THY1* (CD90), and *COL3A1*, the first 2 regarded as genes highly expressed by inflammatory fibroblasts in CD,^22^ were found in the cohort at baseline and were downregulated in patients achieving endoscopic remission with upadacitinib at week 12. In contrast, this downregulation of *THY1* and *COL3A1* was not found in patients treated with anti-TNF therapy. Strikingly, no changes in the expression of genes associated with Th17 or innate lymphocyte cell-3 were found. Additional analysis of the same dataset showed that inflammatory fibroblasts were the most significantly affected cell type in patients responding to upadacitinib treatment.^110^ These data suggest that JAK inhibitors may directly regulate fibroblasts and thereby potentially fibrosis, although the data should be carefully interpreted as no scRNA sequencing data or fibrosis endpoints are available in the CELEST trial. According to a recently published article, scRNA sequencing data from colon biopsies of UC patients treated with tofacitinib showed significant changes in the fibroblast compartment, next to changes in macrophages.^52^ ADAM-like Decysin (ADAMDEC)^+^ fibroblasts, previously identified as colitis-specific fibroblast cluster,^111^ showed the most differentially expressed genes in response to tofacitinib. Importantly, these changes in fibroblast subsets upon JAK inhibition are not directly implicated in fibrosis. Furthermore, since these scRNA sequencing data are obtained from mucosal biopsies, they will mainly contain mucosal fibroblasts. However, these data point to the direction that JAK inhibition also induces their effects beyond immune cells.

Phase 4 post-marketing clinical trials are currently ongoing and will hopefully include patients with signs of active fibrosis and provide some long-term follow-up data on the capability of JAK inhibitors to prevent fibrosis (NCT05930275, NCT06023030). As mentioned before, it will be essential to develop standardized imaging techniques and scoring systems to determine the degree of intestinal fibrosis in these patients. Furthermore, the fact that filgotinib and tofacitinib did not meet their primary clinical endpoints in CD trials does not necessarily mean that there are no (long-term) anti-fibrotic effects of these agents. It could be useful to reevaluate biomaterials from patients who participated in these trials to assess the potential anti-fibrotic effects of these JAK inhibitors.

### 4.2. Novel JAK inhibitors in IBD

JAK inhibitors are rapidly moving ahead in clinical trials for IBD. Ivarmacitinib (SHR0302), a preferential JAK1 inhibitor that, is currently being investigated for both UC and CD. A phase 3 study is ongoing that assesses the therapeutic efficacy and safety of ivarmacitinib in moderately to severely active UC (NCT05181137). A phase 2 trial with ivarmacitinib in CD patients has been completed, but no data are publicly available yet (NCT03677648). **Oncostellae** (OST-122) represents another novel JAK inhibitor, which is a gut-selective JAK3/TYK2 and AMP-activated protein kinase-related protein kinase 5 inhibitor. This drug is currently being developed as an anti-inflammatory and potential anti-fibrotic drug for UC and CD. Although the exact role of JAK3 in the context of fibrosis needs to be defined, preclinical studies have shown that JAK3 inhibition has promising effects in models for renal fibrosis.^112^ A phase 1b/2a study assessed the efficacy of OST-122 in moderate to severe UC patients, and preliminary results indicated that the drug is effective and well tolerated, showing limited systemic absorption (NCT04353791).^113^

At this point, no TYK2 inhibitor is available for IBD. In psoriasis, deucravacitinib is the first approved TYK2 inhibitor.^114^ The phase 2 LATTICE trials investigated the therapeutic efficacy and safety of deucravacitinib in CD and UC. The LATTICE-CD (NCT03599622) was prematurely stopped due to lack of efficacy. In LATTICE-UC (NCT03934216), no significant differences in week 12 clinical remission (primary endpoint) and endoscopic response rates were seen compared to placebo (NCT04877990).^115^ However, other TYK2 inhibitors are still under investigation in IBD. Brepocitinib is a TYK2 inhibitor which also targets JAK1, and is currently being investigated in phase 2 clinical trials to assess efficacy in moderate to severe UC (NCT02958865) and CD (NCT03395184).^116–118^ In a recent study exploring the potential of brepocitinib in cicatricial alopecia, enhanced (but non-significant) gene expression of profibrotic markers, such as collagen (types 1A, 3A, and 4A) and TGF-β, was seen in patients who received placebo compared to patients in the brepocitinib group, suggesting that this agent might prevent fibrosis.^119^ Ritlecitinib, an FDA and EMA-approved JAK3/TEC kinase family inhibitor has also been investigated in the same phase 2b clinical trials as brepocitinib for moderate to severe UC (NCT02958865) and CD (NCT03395184).^118^ This dual inhibitor inhibits the TEC kinase family, next to JAK3, leading to impaired signal transduction in B and T cells. Other TYK2 inhibitors that are being investigated at this moment for their effectiveness in both CD and UC are VTX958 and zasocitinib.

All these novel JAK inhibitors are predominantly being investigated as potential anti-inflammatory drugs for IBD, but only OST-122 was specifically tested as a potential anti-fibrotic drug. Both additional analysis on patient materials from clinical studies assessing fibroblasts subsets and ECM remodeling after JAK inhibitor therapy and studies in which patients with fibrotic/stricturing disease are eligible to participate, are required to elucidate the potential of JAK inhibitors to treat intestinal fibrosis in IBD.

## 5. Conclusions and future perspectives

Initially, JAK inhibitors have mainly been used to target lymphocyte activation by blocking multiple cytokine pathways. However, it is now increasingly being accepted that JAK inhibitors exert broad effects on other cell types, including stromal cells. Since stromal cells, and especially activated fibroblasts, play an essential role in fibrosis formation, JAKs represent a potential target for the treatment of intestinal fibrosis. Here, we highlight the role of the JAK-STAT signaling pathway in intestinal fibrosis and JAK inhibition as a potential anti-fibrotic approach in IBD, also based on their use for other fibrotic-related diseases. Some of the main cytokines that play an important role in intestinal fibrosis, such as TGF-β1 and IL-11, signal directly or indirectly through the JAK-STAT pathway, and more specifically via STAT3. In preclinical models, STAT3 inhibition showed reduced intestinal fibrosis formation. Despite the fact that 3 JAK inhibitors have been approved for UC (ie tofacitinib, filgotinib, and upadacitinib), and one for CD (upadacitinib) in Europe, clinical studies assessing the anti-fibrotic potential of JAK inhibitors in IBD are lacking. Interestingly, available scRNA sequencing data from intestinal biopsies of IBD patients show changes in the stromal compartment upon treatment with JAK inhibitors.

Given the circumferential evidence from both in vitro and in vivo data now available on the potential of JAK inhibition to inhibit intestinal fibrosis, it is time to take the next step. We propose to include patients with intestinal fibrosis in ongoing JAK inhibition post-marketing trials and to follow them with predefined endpoints for fibrosis. It is important to use new imaging tools for fibrosis scoring together with clinical endpoints such as “time to resection or dilatation.” In addition, renewed consensus is needed regarding the nomenclature of fibroblasts in IBD, so that changes in fibroblasts upon treatment are discussed in the same way in the different studies. Hopefully, this will lead to a novel therapeutic approach for IBD patients with an increased risk for stricturing disease receiving timely and efficient top-down treatment with JAK inhibitors.

## Data Availability

No new data are generated.
